# *Passiflora incarnata* attenuation of neuropathic allodynia and vulvodynia apropos GABA-ergic and opioidergic antinociceptive and behavioural mechanisms

**DOI:** 10.1186/s12906-016-1048-6

**Published:** 2016-02-24

**Authors:** Urooj Aman, Fazal Subhan, Muhammad Shahid, Shehla Akbar, Nisar Ahmad, Gowhar Ali, Khwaja Fawad, Robert D. E. Sewell

**Affiliations:** Department of Pharmacy, University of Peshawar, Peshawar, 25120 Khyber Pakhtunkhwa Pakistan; Cardiff School of Pharmacy and Pharmaceutical Sciences, Cardiff University, Cardiff, CF10 3NU UK

**Keywords:** *Passiflora incarnata*, Allodynia, Vulvodynia, GABA receptors, Opioid receptors, Neuropathic pain

## Abstract

**Background:**

*Passiflora incarnata* is widely used as an anxiolytic and sedative due to its putative GABAergic properties. *Passiflora incarnata* L. methanolic extract (PI-ME) was evaluated in an animal model of streptozotocin-induced diabetic neuropathic allodynia and vulvodynia in rats along with antinociceptive, anxiolytic and sedative activities in mice in order to examine possible underlying mechanisms.

**Methods:**

PI-ME was tested preliminary for qualitative phytochemical analysis and then quantitatively by proximate and GC-MS analysis. The antinociceptive property was evaluated using the abdominal constriction assay and hot plate test. The anxiolytic activity was performed in a stair case model and sedative activity in an open field test. The antagonistic activities were evaluated using naloxone and/or pentylenetetrazole (PTZ). PI-ME was evaluated for prospective anti-allodynic and anti-vulvodynic properties in a rat model of streptozotocin induced neuropathic pain using the static and dynamic testing paradigms of mechanical allodynia and vulvodynia.

**Results:**

GC-MS analysis revealed that PI-ME contained predominant quantities of oleamide (9-octadecenamide), palmitic acid (hexadecanoic acid) and 3-hydroxy-dodecanoic acid, among other active constituents. In the abdominal constriction assay and hot plate test, PI-ME produced dose dependant, naloxone and pentylenetetrazole reversible antinociception suggesting an involvement of opioidergic and GABAergic mechanisms. In the stair case test, PI-ME at 200 mg/kg increased the number of steps climbed while at 600 mg/kg a significant decrease was observed. The rearing incidence was diminished by PI-ME at all tested doses and in the open field test, PI-ME decreased locomotor activity to an extent that was analagous to diazepam. The effects of PI-ME were antagonized by PTZ in both the staircase and open field tests implicating GABAergic mechanisms in its anxiolytic and sedative activities. In the streptozotocin-induced neuropathic nociceptive model, PI-ME (200 and 300 mg/kg) exhibited static and dynamic anti-allodynic effects exemplified by an increase in paw withdrawal threshold and paw withdrawal latency. PI-ME relieved only the dynamic component of vulvodynia by increasing flinching response latency.

**Conclusions:**

These findings suggest that *Passiflora incarnata* might be useful for treating neuropathic pain. The antinociceptive and behavioural findings inferring that its activity may stem from underlying opioidergic and GABAergic mechanisms though a potential oleamide-sourced cannabimimetic involvement is also discussed.

**Electronic supplementary material:**

The online version of this article (doi:10.1186/s12906-016-1048-6) contains supplementary material, which is available to authorized users.

## Background

Pain is an unpleasant sensory and emotional experience associated with actual or potential tissue damage or described in terms of such damage [1]. The phenomenon of pain may be nociceptive or neuropathic in nature, and caused by damage to non-neural or neuronal tissues respectively [2, 3]. Neuropathic pain is a major cause of morbidity and has a profound impact on patient well-being. It involves the sensation of allodynia; a painful sensation to a normally non-noxious stimulus and hyperalgesia; an exaggerated pain response to a normally noxious stimulus [4]. Neuropathic pain results from various causes that affect the central nervous system including multiple sclerosis, post stroke or spinal cord pain. Alternatively, it may be associated with damage to the peripheral nervous system, for instance, diabetic neuropathy and trigeminal or post-herpetic neuralgia [5]. Management of neuropathic pain poses an enormous challenge due to the restricted efficacy of assorted pharmacotherapies including both natural treatments [6–8] and synthetic medicaments [9, 10] which are limited by the occurrence of side effects and the extent of pain inhibition [11].

*Passiflora incarnata* L. (Additional file 1: Figure S1) from the genus *Passiflora* (family: Passifloraceae) commonly known as Passion flower, is a fast growing perennial vine widely spread in tropical and warm temperate regions [12]. Phytochemical analysis of *P. incarnata* has demonstrated that flavonoids constitute about 2.5 % of the total phyto-constituents [13, 14] mainly present in the leaves, the greatest concentration of flavonoid being vitexin compared to the other species of its genus [12, 15]. *P. incarnata* has been studied for its analgesic [16], anxiolytic [17–20], anticonvulsant [21], antitussive [22], aphrodisiac [23], anti-asthmatic [24], anti-diabetic and hypolipidemic properties [25] along with efficacy in the treatment of cannabinoid [26], morphine [27], nicotine [28] and alcohol dependence [29]. Traditionally, *P. incarnata* has been used for curing various ailments like anxiety, insomnia, convulsions, sexual dysfunction, cough and cancer [30] and is well known in relieving neuropathic conditions [12]. In this regard, an eye wipe test has been conducted suggesting a potential application in relieving trigeminal neuralgia [31]. Clinical investigations on *P. incarnata* have indicated effectiveness in the treatment of anxiety [32, 33], insomnia [34], opioid withdrawal [35], attention deficit hyperactivity disorder [36] and postmenopausal symptoms [37].

Neuropathic pain results from a cascade of neurobiological events that induces electrical hyperexcitability in somatosensory conduction pathways and results in hyperesthesia, dysesthesia, hyperalgesia, paresthesia or allodynia [38]. Currently, the most common choices of therapy for neuropathic pain are tricyclic antidepressants and anticonvulsants [39, 40]. However, these therapies are only partially effective and are usually accompanied by a variety of side effects [41]. The use of complementary and alternative medicine has been shown to produce some beneficial effects in the management of painful neuropathy [42] and several herbal medicines exhibit promise in different types of experimentally induced neuropathic pain models [6, 8, 43–45]. Thus there is some scope for new herbal medicines to combat neuropathic pain syndromes [46]. The present study was therefore designed to evaluate the ameliorative effect of *P. incarnata* methanolic extract (PI-ME) in an animal model of streptozotocin-induced diabetic neuropathic allodynia and vulvodynia [47] in rodents. Additionally, PI-ME induced antinociceptive, anxiolytic and sedative activities were also investigated using naloxone and pentylenetetrazole (PTZ) to probe its possible underlying mechanisms.

## Methods

### Chemicals

Morphine (Punjab Drug House, Lahore, Pakistan), diclofenac sodium (≥98 %, Continental Chemicals Company Pvt. Ltd. Pakistan), naloxone (98 %, Hangzhou Uniwise International Co., Ltd, China), gabapentin (99 %, MKB Pharmaceuticals Pvt Ltd Peshawar, Pakistan), diazepam (Valium 10 mg/ 2 ml, Roche, Pakistan), pentylenetetrazole (≥98 %, Sigma Aldrich, UK) , streptozotocin (≥98 %, Sigma Aldrich, UK) and commercial grade methanol (Haq Chemicals Ltd Peshawar, Pakistan).

### Preparation of *Passiflora incarnata* methanolic extract

*P. incarnata* whole plant was collected from the botanical garden of the Department of Pharmacy, University of Peshawar. It was authenticated by Prof. Dr. Mohammad Ibrar of the Department of Botany, University of Peshawar and a specimen was deposited in the herbarium with a voucher number 20062 (PUP). The aerial parts were separated, shade dried, and coarsely powdered (1000 g). It was macerated for 7 days with commercial grade methanol (5 L). The extract was filtered and concentrated under reduced pressure at 60 °C in a rotary evaporator until a semisolid extract containing no methanol was obtained (yield: 31.20 %).

### Phytochemical analysis

PI-ME was preliminary evaluated by qualitative phytochemical analysis [47] and was further screened by quantitative analysis of flavonoids, alkaloids, saponins and tannins [48, 49]. It was also subjected to gas chromatography/mass spectrometry (GC/MS) analysis which was carried out on a 6890 N Agilent gas chromatograph coupled to a JMS 600 H JEOL mass spectrometer. The compound mixture was separated on a fused silica capillary SPBI column, 30 m × 0.32 mm, 0.25 μm film thickness, in a temperature program from 50 to 256 °C with a rate of 4 °C/min with 2 min hold. The injector was at 260 °C and the flow rate of the carrier gas, helium was 1 mL/min. The EI mode of the JMS 600 H JEOL mass spectrometer had an ionization voltage of 70 eV, electron emission of 100 μA, ion source temperature of 250 °C and analyzer temperature of 250 °C. Samples were injected manually in split mode and the total elution time was 90 min. MS scanning was performed from m/z 85 to 390. Identification of the active constituents was based on the computer evaluation of mass spectra of the sample through NIST-based AMDIS (automated mass spectral deconvolution and identification software), direct comparison of peaks and retention times with those of standard compounds as well as by following the characteristic fragmentation patterns of the mass spectra of particular classes of compounds.

### Animals

BALB/c mice (18–26 g) and female Sprague Dawley rats (150-200 g) maintained in a 12 h light/dark cycle at 22 ± 2 °C were used in the experiments. Food and water were provided *ad libitum*. Experiments on animals were performed in accordance with the UK Animals (Scientific Procedures) Act 1986 and according to the rules and ethics set forth by the Ethical Committee of the Department of Pharmacy, University of Peshawar. Approval for this study was granted with the registration number: 06/EC-14/Pharm (dated: April 06, 2014). The animal control groups used in experiments were given normal saline which was also the vehicle for all the drugs administered throughout all the experiments.

### Abdominal constriction assay

BALB/c mice (18–22 g, *n* = 8 mice per group) of either sex were injected with 0.6 % acetic acid (10 mL/kg, i.p) to induce an abdominal constriction response [50, 51]*.* In the abdominal constriction assay, the mean incidence of constrictions expressed as % protection across all experiments was normalized relative to untreated controls. PI-ME (150, 200 and 250 mg/kg, p.o), morphine (5 mg/kg, i.p) or diclofenac (50 mg/kg, i.p) were administered 30 min before acetic acid injection. In the opioid antagonism study, the animals were pretreated with naloxone (0.5 mg/kg, s.c), 5 min before acetic acid administration. Percentage protection was calculated as:

$$ \%\ \mathrm{Protection} = \left(1\ \hbox{--}\ \mathrm{Number}\ \mathrm{of}\ \mathrm{abdominal}\ \mathrm{constrictions}\ \mathrm{after}\ \mathrm{treatment}/\mathrm{Number}\ \mathrm{of}\ \mathrm{abdominal}\ \mathrm{constrictions}\ \mathrm{of}\kern0.5em \mathrm{untreated}\kern0.5em \mathrm{control}\right) \times 100 $$

### Hot plate test

BALB/c mice (18–22 g, *n* = 8 mice per group) of either sex were pretested for their response latencies on a hot plate (Harvard apparatus, USA) maintained at 54.0 ± 0.1 °C. The response end-point was signified by hind limb flick, lick or jumping at which point animals were immediately removed from the thermal nociceptive stimulus in order to avoid any tissue damage or possibility of subsequent hyperalgesia. A cut-off time of 30 s was imposed such that if they did not respond within this latency period then they were immediately removed from the hot plate stimulus [51]. Thirty minutes after pretesting, the animals were administered PI-ME (100, 150, 200 mg/kg; p.o), morphine (5 mg/kg; i.p) or diclofenac (50 mg/kg, i.p). In the antagonism studies, naloxone (1.0 mg/kg, s.c) or PTZ (10 mg/kg, i.p) were administered 10 or 30 min respectively before treatment and the animal response latencies were measured at 30, 60, 90 and 120 min. The percentage antinociception was calculated as:

$$ \%\ \mathrm{Antinociception} = \left(\mathrm{Test}\ \mathrm{latency}\ \hbox{--}\ \mathrm{control}\ \mathrm{latency}\right)/\ \left(\mathrm{Cut}\hbox{-} \mathrm{off}\ \mathrm{time}\ \hbox{--}\ \mathrm{control}\ \mathrm{latency}\right) \times 100 $$

### Anxiolytic activity (Staircase test)

BALB/c mice (18–24 g, *n* = 8 mice per group) of either sex were administered PI-ME (200, 400 and 600 mg/kg, p.o) or diazepam (2 mg/kg, i.p). In the drug combination experiments, PTZ (10 mg/kg, i.p) was administered 30 min prior to drug treatment. The number of rears and steps climbed by each animal was observed for 3 min using the staircase apparatus and the methods described by Simiand and coworkers [52]. A step was considered to be climbed only if the criterion was met whereby an animal placed all four paws on the step.

### Locomotor activity

BALB/c mice (18–26 g, *n* = 6 mice per group) of either sex were administered with PI-ME (200, 400 and 600 mg/kg, p.o) or diazepam (4 mg/kg, i.p). In the drug combination experiments, PTZ (10 mg/kg, i.p) was administered 30 min prior to drug treatment. Thirty min later, the animals were placed in the recording apparatus with a floor area of 50 × 40 cm divided into four equal quadrants by lines. The number of lines crossed by each animal was recorded for 30 min using a digital camera (Cat’s Eye IR IP Camera, Taiwan) [53].

### Streptozotocin induced neuropathic pain

#### Induction of mechanical allodynia and vulvodynia

Female Sprague Dawley rats (150– 200 g, *n* = 6 rats per group) food withdrawn for 16 h were administered streptozotocin (50 mg/kg, i.p) and food was immediately provided. On the 5^th^ day, animals exhibiting random blood glucose levels greater than 270 mg/dl were included in the study [54]. Body weights and blood glucose were measured at specified time periods. The bedding material was frequently changed to avoid any infection due to excessive urination. On the 29^th^ day post streptozotocin administration, animals were transferred to wire mesh cages and acclimatized for 15–45 min. They were then assessed for mechanical allodynia or vulvodynia before and after PI-ME or standard gabapentin administration using the von Frey up-down method [55].

### Treatment schedule

Animals were divided into five groups. Group I received normal saline and served as control. Group II remained as the streptozotocin positive control group. Group III received a single intraperitoneal dose of gabapentin (75 mg/kg) and served as the standard. Group IV and V were treated with PI-ME at doses of 200 and 300 mg/kg respectively. The therapeutic doses of PI-ME for evaluation in neuropathic pain were selected on the basis of its analgesic, anxiolytic, locomotor and respective antagonistic activities.

### Assessment of static and dynamic allodynia

Static allodynia was assessed using a series of von Frey filaments (0.4, 0.6, 1, 1.4, 2, 4, 6, 8, 10, 15 g), starting with a 2.0 g force applied perpendicularly to the plantar surface of the right hind paw for 5 s or until the animal displayed a withdrawal response (lifting of the paw). Animals responding to 3.63 g force or below were included in the study and 15 g was selected as the cut-off force [54].

Dynamic allodynia was assessed by lightly stroking the plantar surface of the hind paw with a cotton bud. Lifting or licking the paw was considered as a withdrawal response and the time taken to show a withdrawal reaction was considered as the paw withdrawal latency (PWL). Animals responding to the cotton bud within 8 s were included in the study and 15 s was selected as the cut off time [54].

### Assessment of static and dynamic vulvodynia

Static vulvodynia was assessed by shaving the anogenital area including the mons pubis. A series of von Frey filaments (0.008, 0.02, 0.04, 0.07, 0.16, 0.4, 0.6, 1 g), were applied perpendicularly to the mucous membrane of the anogenital region for 4 s starting with a 0.04 g force, until a flinching response occurred. Animals responding to a 0.16 g force or below were included in the study and a 1.0 g force was selected as the cut-off force [56].

Dynamic vulvodynia was assessed by lightly brushing a cotton bud over the mucous membrane of the anogenital region for 10 s or until a flinching response occurred. Animals showing a flinching response within 5 s were included in the study and 10 s was selected as the cut-off time [56].

### Statistical analysis

Data were expressed as mean ± SEM. Statistical comparisons were carried out by one way ANOVA followed by Dunnett’s, Bonferroni or Tukey’s multiple comparison tests where appropriate using GraphPad Prism 5 (GraphPad Software Inc. San Diego CA, USA). Statistical significance was deduced at *P* ≤ 0.05.

## Results

### Phytochemical analysis of *Passiflora incarnata*

**Fig 1:**
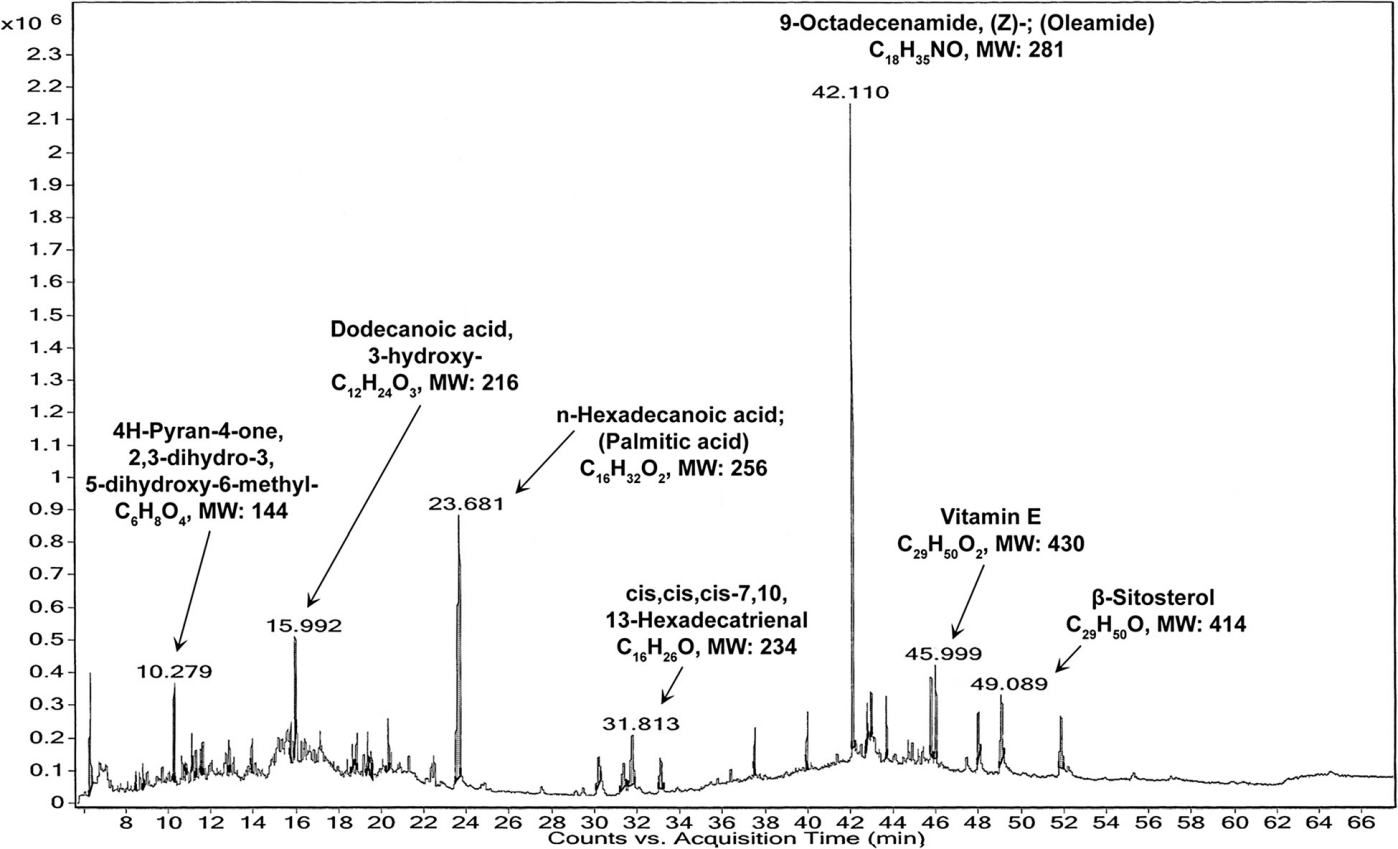
MS chromatogram of *Passiflora incarnata* methanolic extract

Preliminary qualitative analysis of PI-ME disclosed the presence of flavonoids, alkaloids, carbohydrates, tannins, glycosides, fixed oils and saponins (Table 1). Subsequent more detailed quantitative analysis revealed the presence of flavonoids (72 %), saponins (10 %) and alkaloids (13.4 %) in PI-ME. The major compounds obtained from GC-MS analysis of PI-ME included: 9-Octadecenamide (Oleamide) (C_18_H_35_NO, MW: 281), n-Hexadecanoic acid (Palmitic acid) (C_16_H_32_O_2_, MW: 256), dodecanoic acid, 3-hydroxy- (C_12_H_24_O_3_, MW: 216), 4H-Pyran-4-one, 2,3-dihydro-3,5-dihydroxy-6-methyl- (C_6_H_8_O_4_, MW: 144), vitamin-E (C_29_H_50_O_2_, MW: 430), cis,cis,cis-7,10,13-Hexadecatrienal (C_16_H_26_O, MW: 234), β-Sitosterol (C_29_H_50_O, MW: 414), 9,10-Secocholesta-5,7,10(19)-triene-3,24,25-triol, (3β,5Z,7E)- (C_27_H_44_O_3_, MW: 416), pregnane-3,11,20,21-tetrol, cyclic 20,21-(butyl boronate), (3α,5β,11β,20R)- (C_25_H_43_BO_4_, MW: 418), ethyl 9-hexadecenoate (C_18_H_34_O_2_, MW: 282), stigmasterol (C_29_H_48_O, MW: 412), octadecanoic acid (C_18_H_36_O_2_, MW: 284), 2H-1-Benzopyran-6-ol, 3,4-dihydro-2,8-dimethyl-2- (4,8,12-trimethyltridecyl)-, [2R-[2R*(4R*,8R*)]]- (C_27_H_46_O_2_, MW: 402), and phytol (C_20_H_40_O, MW: 296) among other important constituents (Table 2 and Fig. 1).

**Table 1:** Preliminary qualitative phytochemical analysis of *Passiflora incarnata* methanolic extract (PI-ME)

Sample	Test	Observation	Result
1.	Aqueous solution of PI-ME + 10 % ammonium hydroxide solution	Appearance of yellow coloration	Flavonoids present
2.	A portion of PI-ME + few drops of Wagner’s reagent	Reddish brown precipitate	Alkaloids present
3.	A small volume of PI-ME + 1–2 drops of Mayer’s reagent	Creamy or white precipitate	Alkaloids present
4.	0.5 ml PI-ME + 0.5 ml benedict’s reagent → mixed and boiled for 2 min	Characteristic colored precipitate	Carbohydrates present
5.	1 ml PI-ME + 1 ml Barfoed’s reagent → boiled for 2 min	Red precipitate	Carbohydrates present
6.	50 mg PI-ME + 5 ml distilled water + small amount of 5 % ferric chloride solution	Intense green coloration	Tannins and phenolic compounds present
7.	50 mg PI-ME + conc. HCL → heated on water bath for 2 h → resultant hydrolysate filtered → 2 ml hydrolysate + 3 ml chloroform → chloroform layer separated out + 10 % ammonia solution	Pink coloration	Glycosides present
8.	A small amount of PI-ME → compressed between two pieces of filter paper	Formation of oil spot on filter paper	Fixed oils present
9.	50 mg PI-ME + 20 ml distilled water → shaken for 15 min	Formation of 2 cm thick layer of foam	Saponins present

**Table 2:** GC/MS analysis of *Passiflora incarnata* methanolic extract

Chemical constituent	Formula	Molecular weight	R.T. (min)	Percent abundance
10-Undecen-1-al, 2-methyl-	C_12_H_22_O	182	8.465	0.377
1,3-Pentanediamine	C_5_H_14_N_2_	102	8.809	0.353
4H-Pyran-4-one, 2,3-dihydro-3,5-dihydroxy-6-methyl-	C_6_H_8_O_4_	144	10.27	5.477
1-Pentanol, 2-methyl-, acetate	C_8_H_16_O_2_	144	10.83	0.621
1,2,6-Hexanetriol	C_6_H_14_O_3_	134	11.10	0.623
4-Cyclopropylcarbonyloxytridecane	C_17_H_32_O_2_	268	11.32	0.664
5-Cyclopropylcarbonyloxypentadecane	C_19_H_36_O_2_	296	11.60	0.796
9-Tetradecen-1-ol, acetate, (E)-	C_16_H_30_O_2_	254	12.87	0.488
trans-2-undecenoic acid	C_11_H_20_O_2_	184	15.98	0.438
Dodecanoic acid, 3-hydroxy-	C_12_H_24_O_3_	216	15.99	13.64
4-((1E)-3-Hydroxy-1-propenyl)-2-methoxyphenol	C_10_H_12_O_3_	180	18.64	0.393
d-Mannose	C_6_H_12_O_6_	180	18.86	0.378
7-Methyl-Z-tetradecen-1-ol acetate	C_17_H_32_O_2_	268	19.34	0.395
l-Gala-l-ido-octose	C_8_H_16_O_8_	240	19.54	0.406
n-Hexadecanoic acid; (Palmitic acid)	C_16_H_32_O_2_	256	23.68	21.86
Phytol	C_20_H_40_O	296	30.20	1.004
9-Hexadecyn-1-ol	C_16_H_30_O	238	31.33	0.956
cis,cis,cis-7,10,13-Hexadecatrienal	C_16_H_26_O	234	31.81	2.175
Octadecanoic acid	C_18_H_36_O_2_	284	33.09	1.209
9-Octadecenamide, (Z)-; (Oleamide)	C_18_H_35_NO	281	42.11	33.52
9,10-Secocholesta-5,7,10(19)-triene-3,24,25-triol, (3β,5Z,7E)-	C_27_H_44_O_3_	416	42.79	1.762
Pregnane-3,11,20,21-tetrol, cyclic 20,21-(butyl boronate), (3α,5β,11β,20R)-	C_25_H_43_BO_4_	418	42.98	1.422
2H-1-Benzopyran-6-ol, 3,4-dihydro-2,8-dimethyl-2-(4,8,12-trimethyltridecyl)-, [2R-[2R*(4R*,8R*)]]-	C_27_H_46_O_2_	402	43.69	1.033
Ethyl 9-hexadecenoate	C_18_H_34_O_2_	282	45.78	1.390
Vitamin E	C_29_H_50_O_2_	430	45.99	2.579
Stigmasterol	C_29_H_48_O	412	48.01	1.229
β-Sitosterol	C_29_H_50_O	414	49.08	1.776

### Antinociceptive activity of *Passiflora incarnata*

#### Abdominal constriction assay (tonic visceral chemically-induced nociception)

**Fig. 2:**
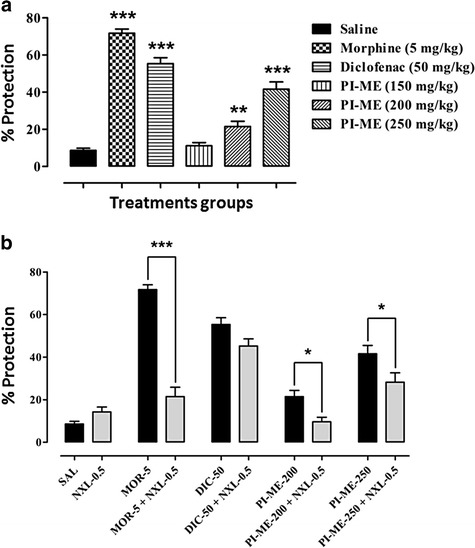
**a** Antinociceptive activity of *Passiflora incarnata* (PI-ME) in the mouse abdominal constriction assay. ***P < 0.001, **P < 0.01 compared to saline vehicle control (ANOVA followed by Dunnett’s *post hoc* test), (*n* = 8 mice per group). **b** Effect of naloxone (NXL-0.5) on the antinociceptive activity of PI-ME in the mouse abdominal constriction assay. *P < 0.05, ***P < 0.001 compared to morphine (MOR-5), diclofenac (DIC-50) or PI-ME (ANOVA followed by Bonferroni’s multiple comparison *post hoc* test), (*n* = 8 mice per group)

A significant attenuation (*F*_(5,42)_ = 91.99, *P* < 0.001) of acetic acid incited abdominal constriction was produced by PI-ME at doses of 200 mg/kg (*P* < 0.01) and 250 mg/kg (*P* < 0.001) compared to saline control. Similarly, a significant increase (*P* < 0.001) in the percentage protection against abdominal constriction was observed with diclofenac (50 mg/kg) and morphine (5 mg/kg) (Fig. 2a). Naloxone (0.5 mg/kg) (*F*_(9,70)_ = 44.75, *P* < 0.001) significantly reversed the antinociceptive activity of morphine (*P* < 0.001) and PI-ME (200 and 250 mg/kg) (*P* < 0.05) but not that of diclofenac (50 mg/kg) as shown in Fig. 2b.

#### Hot plate test (acute phasic thermal nociception)

**Fig. 3:**
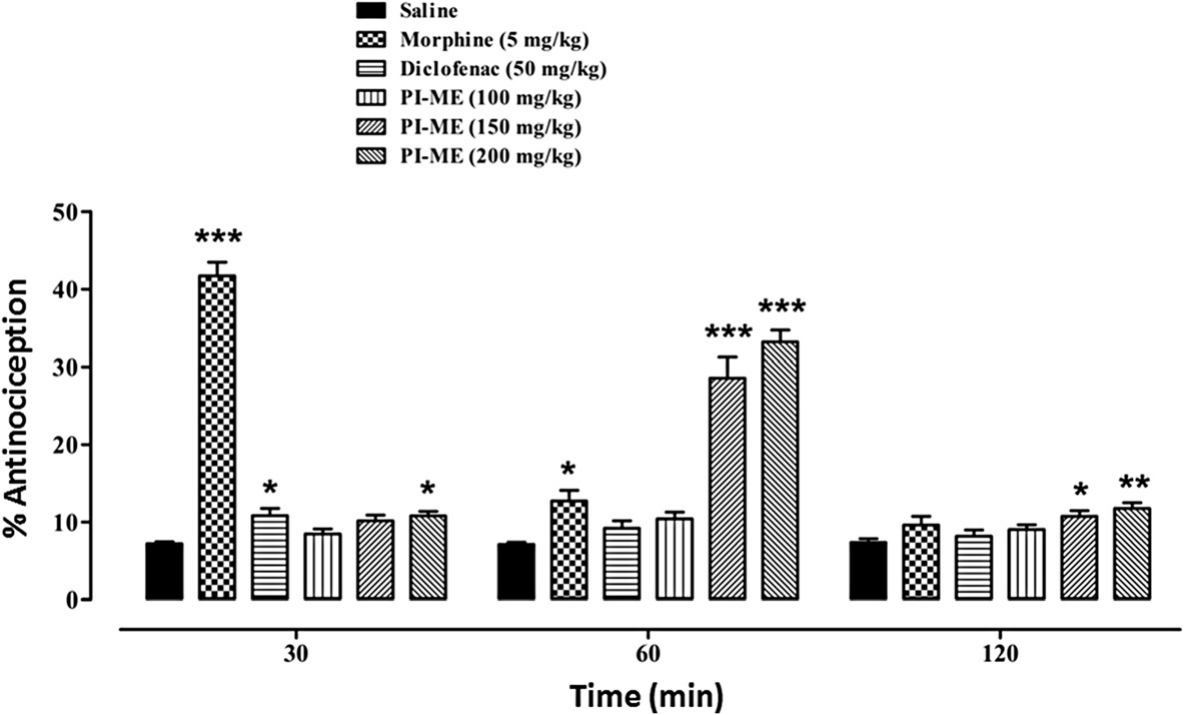
Antinociceptive activity of *Passiflora incarnata* (PI-ME) in the mouse hot plate test. *P < 0.05, **P < 0.01, ***P < 0.001 compared to saline vehicle control (ANOVA followed by Dunnett’s *post hoc* test), (*n* = 8 mice per group)

**Fig. 4:**
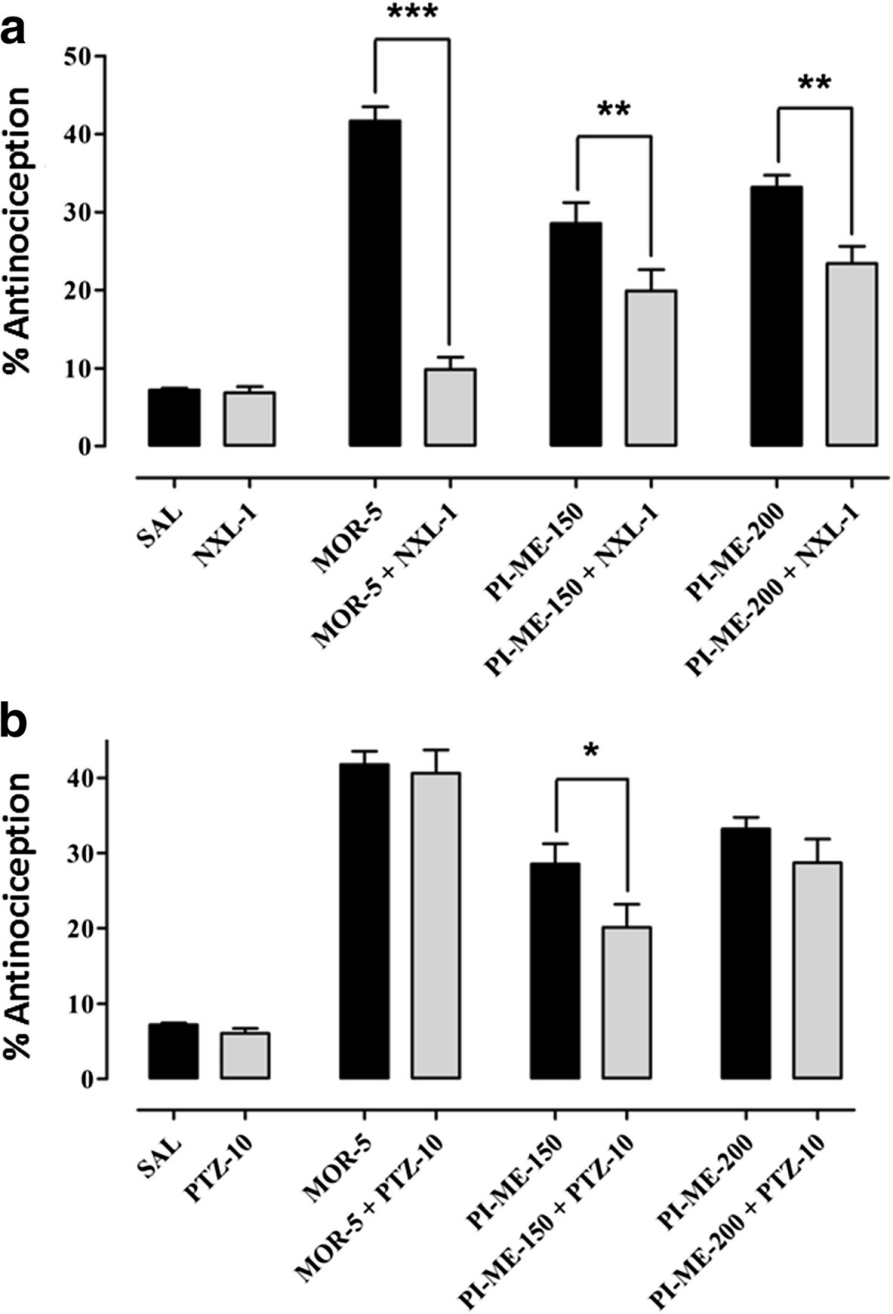
**a** Effect of naloxone (NXL-1); or **b** pentylenetetrazole (PTZ-10) on the antinociceptive effect of *Passiflora incarnata* (PI-ME) in the mouse hot plate test. *P < 0.05, **P < 0.01, ***P < 0.001 compared to morphine (MOR-5) or PI-ME (150 or 200 mg/kg) (ANOVA followed by Bonferroni’s multiple comparison *post hoc* test), (*n* = 8 mice per group)

In the hot plate test, 30 min after drug administration (*F*_(5,42)_ = 200.2, *P* < 0.001) a marked increase in percentage antinociception was observed with morphine (5 mg/kg) (*P* < 0.001), diclofenac (50 mg/kg) (*P* < 0.05) and PI-ME at a dose of 200 mg/kg (*P* < 0.05). After 60 min (*F*_(5,42)_ = 55.36, *P* < 0.001), the increase in percentage response was less significant (*P* < 0.05) for morphine whilst it was highly significant (*P* < 0.001) for PI-ME (150 and 200 mg/kg), the activity being retained in the latter case up to 90 min (*F*_(5,42)_ = 36.61, *P* < 0.001, not shown). However, after 120 min (*F*_(5,42)_ = 4.352, *P* < 0.01) it was only PI-ME at doses of 150 mg/kg (*P* < 0.05) and 200 mg/kg (*P* < 0.01) that afforded protection against thermal nociception (Fig. 3). Naloxone (1.0 mg/kg) (*F*_(7,56)_ = 46.60, *P* < 0.001) reduced the % antinociceptive effect of both morphine (*P* < 0.001) and PI-ME (150 and 200 mg/kg) (*P* < 0.01) (Fig. 4a). Pentylenetetrazole (10 mg/kg) (*F*_(7,56)_ = 35.91, *P* < 0.001) by way of contrast, significantly reduced the antinociceptive effect of PI-ME only at the 150 mg/kg dose (*P* < 0.05) (Fig. 4b).

### Anxiolytic-like activity of *Passiflora incarnata*

**Fig. 5:**
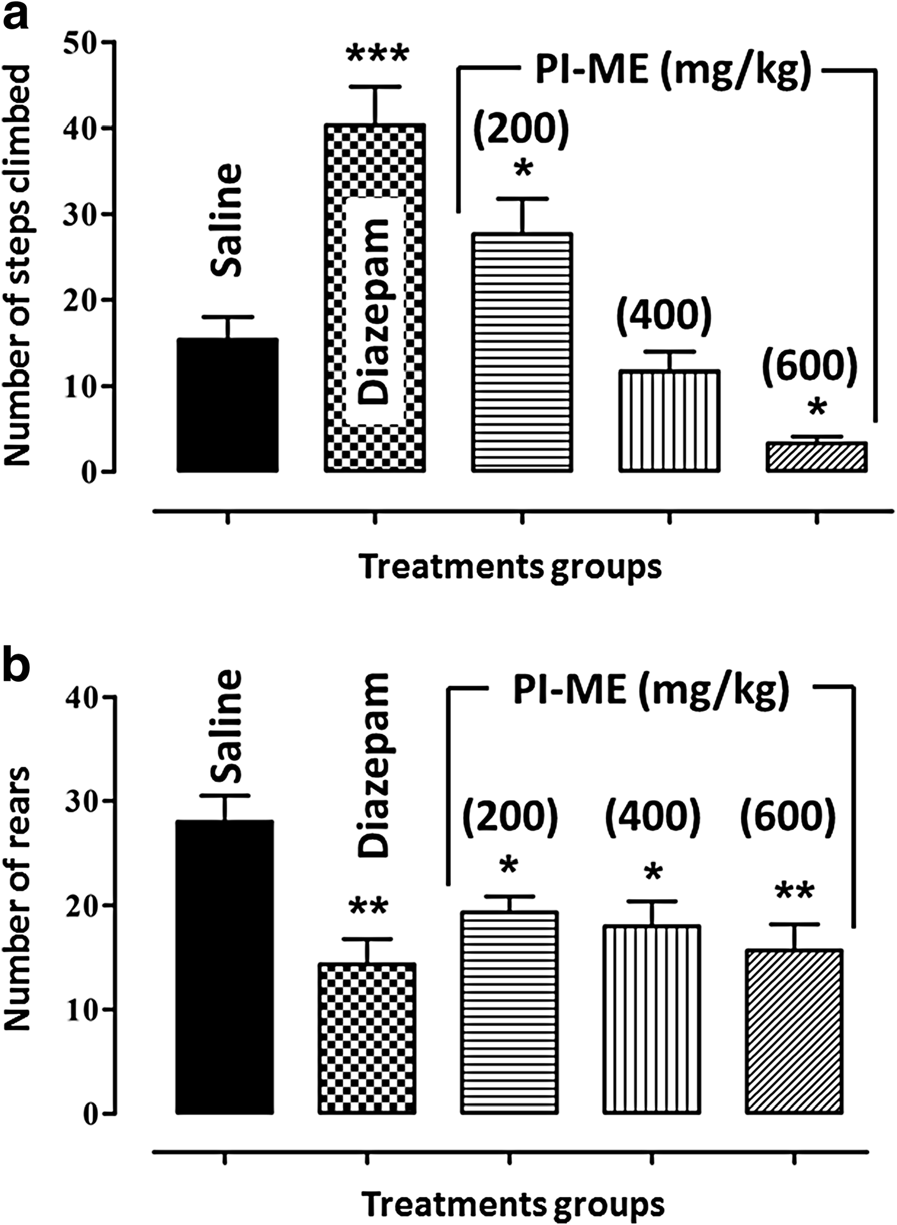
Effect of *Passiflora incarnata* (PI-ME), and diazepam (2 mg/kg) on **a** the number of steps climbed in the staircase test and **b** the incidence of rears in mice. *P < 0.05, **P < 0.01, ***P < 0.001 compared to saline vehicle control (ANOVA followed by Dunnett’s *post hoc* test), (*n* = 8 mice per group)

**Fig. 6:**
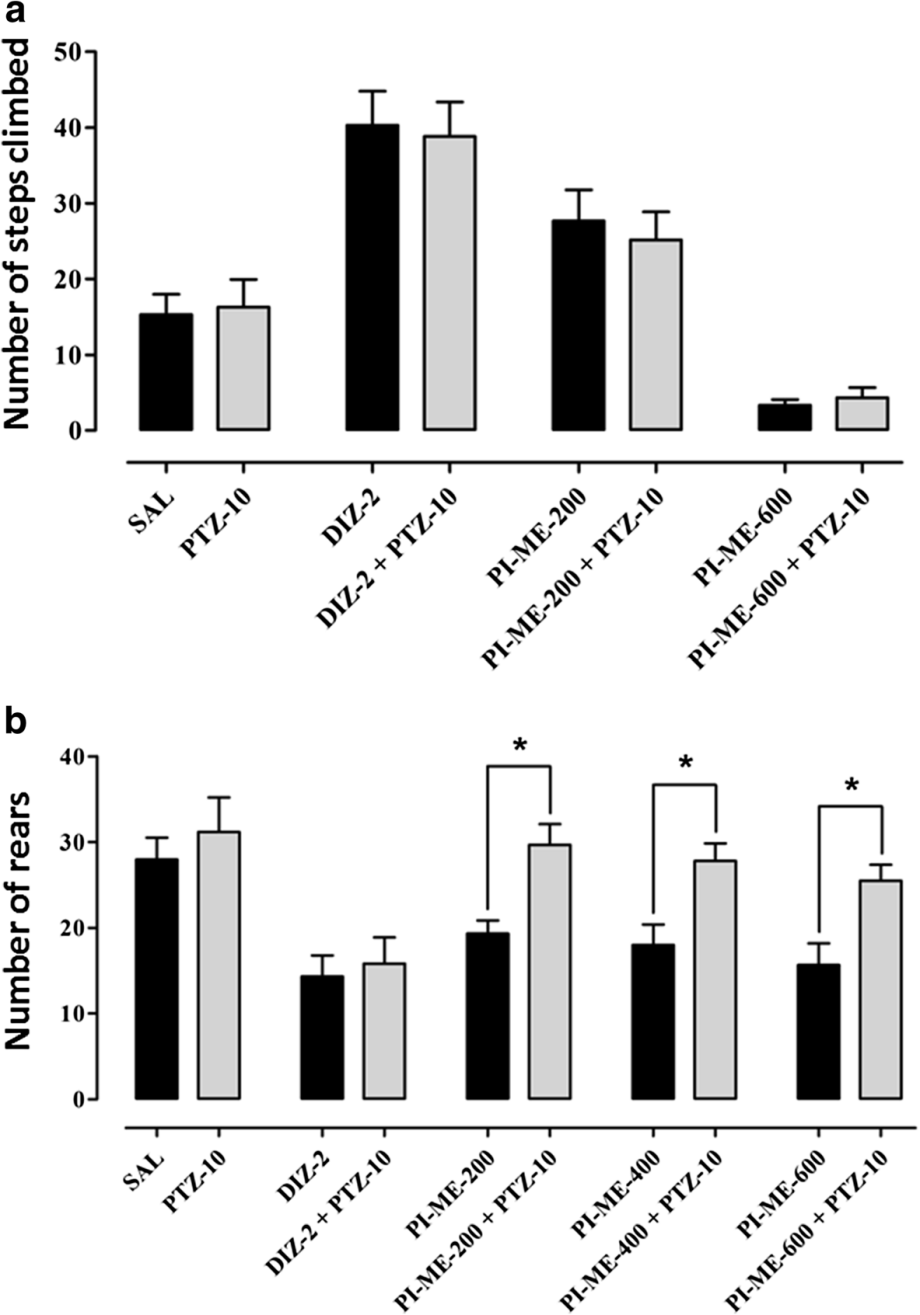
Effect of pentylenetetrazole (PTZ-10) on diazepam (DIZ-2) or *Passiflora incarnata* (PI-ME, 200 and 600 mg/kg) with respect to **a** the number of steps climbed in the staircase test and **b** the incidence of rears in mice. *P < 0.05 compared to PI-ME alone (200, 400 or 200 mg/kg) (ANOVA followed by Dunnett’s *post hoc* test), (*n* = 8 mice per group)

In the staircase test, there was a substantial increase in the number of steps climbed (*F*_(4,25)_ = 21.04, *P* < 0.001) in response to both diazepam (2 mg/kg, *P* < 0.001) and PI-ME (200 mg/kg, *P* < 0.05) versus the animal control group treated with saline vehicle. However, at the highest dose (600 mg/kg) the passiflora extract significantly reduced (*P* < 0.05) the number of steps climbed in comparison with the controls (Fig. 5a). In contrast, the number of rears (*F*_(4,25)_ = 5.403, *P* < 0.01) was inhibited not only by treatment with diazepam (*P* < 0.01) but also by all three doses of PI-ME (200 and 400 mg/kg, *P* < 0.05; 600 mg/kg, *P* < 0.01) in comparison with the saline vehicle controls (Fig. 5b). The *post hoc* test revealed that there was no significant effect of pentylenetetrazole (10 mg/kg) by itself on step climbing nor was there any modification of the stair climbing responses when it was administered in combination with diazepam or PI-ME (Fig. 6a). However, it did reverse the decrement in rears initiated by PI-ME (200, 400 and 600 mg/kg) and actually augmented (*P* < 0.05) the overall rearing incidence (*F*_(9,50)_ = 6.497, *P* < 0.001) as shown in Fig. 6b.

### Sedative activity of *Passiflora incarnata*

#### Locomotor activity

**Fig. 7:**
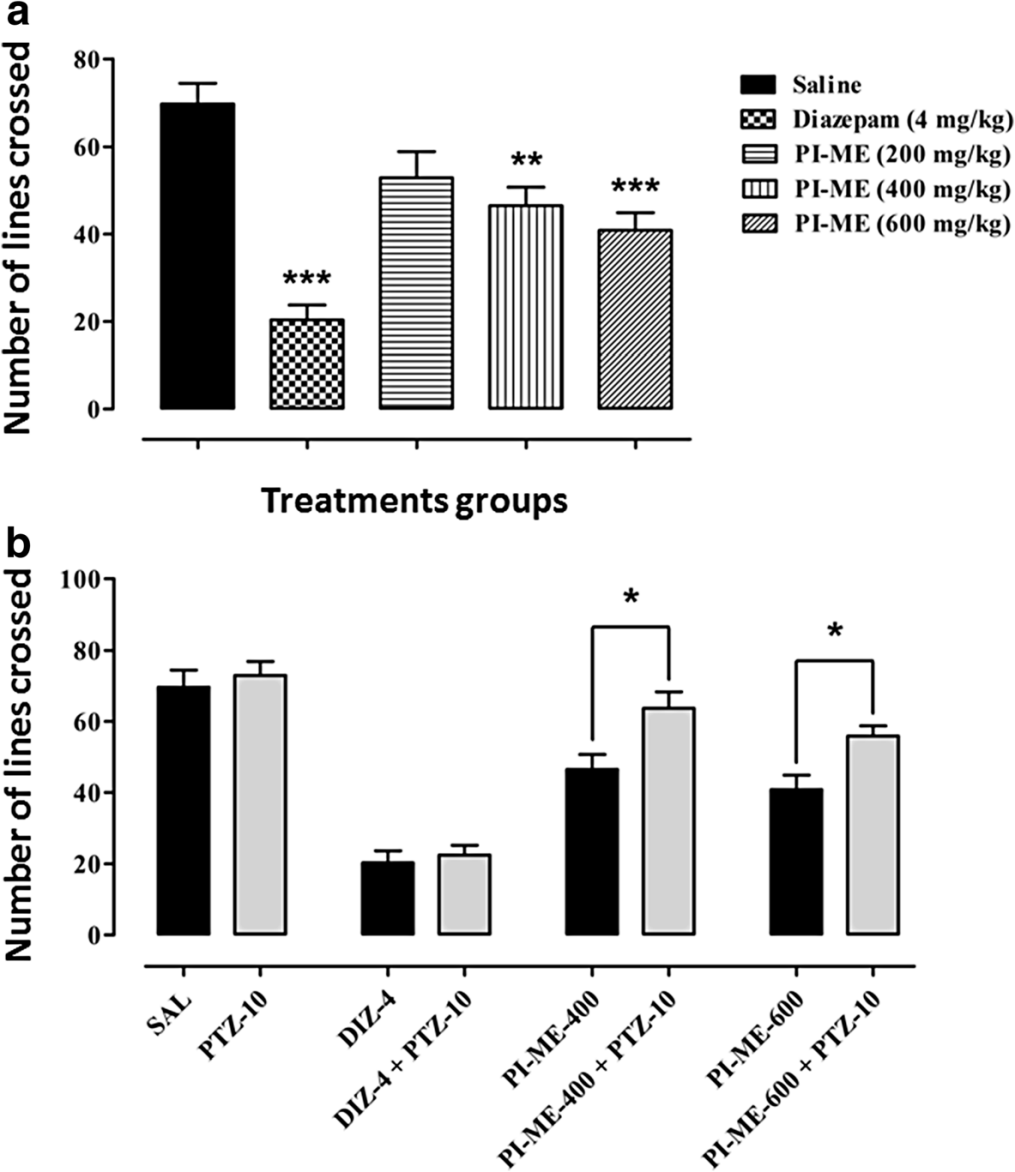
**a** Effect of diazepam and *Passiflora incarnata* (PI-ME) on mouse locomotor activity. **P < 0.01, ***P < 0.001 compared to saline vehicle control (ANOVA followed by Dunnett’s *post hoc* test), (*n* = 6 mice per group). **b** Effect of pentylenetetrazole (PTZ-10) on the mouse locomotor activity induced by *Passiflora incarnata* (PI-ME 400 and 600 mg/kg) or diazepam (DIZ-4). *P < 0.05 compared to PI-ME (400 or 600 mg/kg) alone (ANOVA followed by Bonferroni’s multiple comparison *post hoc* test), (*n* = 6 mice per group)

In the locomotor activity study, there was a pronounced reduction in cage line crossing instigated by both (*F*_(4,25)_ = 15.39, *P* < 0.001) diazepam (4.0 mg/kg, *P* < 0.001) and PI-ME at 400 mg/kg (*P* < 0.01) and 600 mg/kg (*P* < 0.001) though there was no significant motoric decline at the lowest PI-ME dose (200 mg/kg, *P* > 0.05) (Fig. 7a). Pentylenetetrazole (10 mg/kg) (*F*_(7,40)_ = 26.88, *P* < 0.001) blocked (*P* < 0.05) the reduced locomotor effect of PI-ME (400 and 600 mg/kg) by increasing the incidence of line crossing but it did not modify the diazepam locomotor regression (Fig. 7b).

### Effect of *Passiflora incarnata* on mechanical allodynia and vulvodynia

**Fig. 8:**
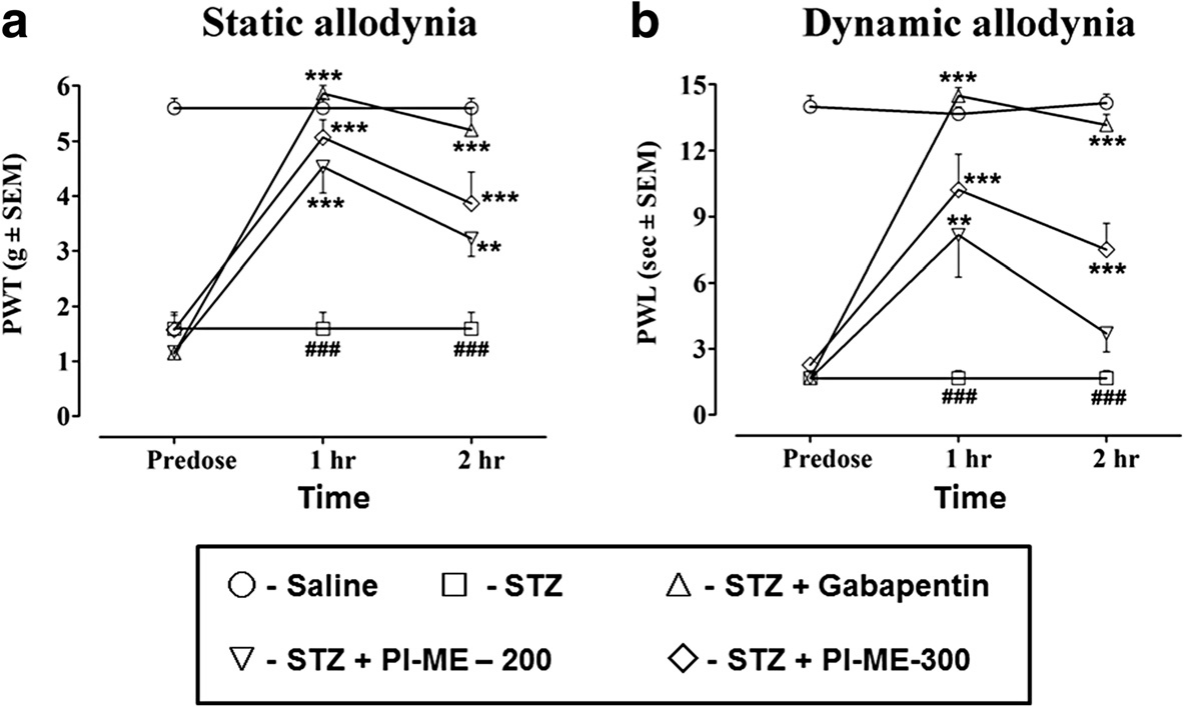
Effect of *Passiflora incarnata* (PI-ME) and gabapentin on **a** static or **b** dynamic allodynia, at 1 or 2 h post-treatment times in female rats on day 29 in a streptozotocin (STZ) induced neuropathic pain model. ^###^ *P* < 0.001 compared to saline control, *P < 0.01, **P < 0.05, ***P < 0.001 compared to streptozotocin alone treated animals, (*n* = 6 rats per group)

Animals administered a single streptozotocin (50 mg/kg) treatment developed both static and dynamic allodynia in their hind paws when tested 29 days later (Fig. 8). Hence, there was a substantial decrease (*P* < 0.001) in PWT and PWL in comparison with saline treated animals. One hour after PI-ME dosing in STZ-pretreated animals on the test day, there was an ensuing increase in PWT (*F*_(4,25)_ = 31.41, *P* < 0.001) and PWL (*F*_(4,25)_ = 20.25, *P* < 0.001) observed for PI-ME at doses of 200 mg/kg (*P* < 0.001, *P* < 0.01) and 300 mg/kg (*P* < 0.001). Similarly, 2 h following treatment with PI-ME on the test day in the STZ-pretreated group, there was a sizeable increase in PWT (*F*_(4,25)_ = 17.92, *P* < 0.001) noted at doses of 200 mg/kg (*P* < 0.01) and 300 mg/kg (*P* < 0.001) of PI-ME. However, at the 2 h test, PWL (*F*_(4,25)_ = 59.63, *P* < 0.001) was increased only by the 300 mg/kg (*P* < 0.001) rather than the 200 mg/kg PI-ME dose. Gabapentin (75 mg/kg) administered as a positive control, also generated an alleviation of mechanical allodynia by elevating (*P* < 0.001) PWT and PWL compared to the 29-day streptozotocin alone pretreated animals at both the 1 and 2 h testing times (Fig. 8).

**Fig. 9:**
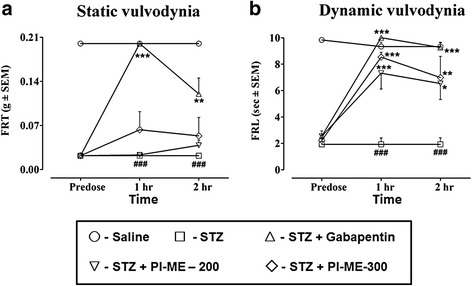
Effect of *Passiflora incarnata* (PI-ME) and gabapentin on **a** static or **b** dynamic vulvodynia at 1 or 2 h post-treatment times in female rats on day 29 in the streptozotocin (STZ) induced neuropathic pain model. ^###^ *P* < 0.001 compared to saline, *P < 0.01, **P < 0.05, ***P < 0.001 compared to streptozotocin alone treated animals, (*n* = 6 rats per group)

The animal group pretreated with streptozotocin by itself 29 days earlier expressed mechanical static and dynamic vulvodynia (*P* < 0.001) compared to the saline vehicle treated controls on the test day (Fig. 9). It was notable that PI-ME (200 and 300 mg/kg) did not modify the diminished FRT (streptozotocin induced static vulvodynia) at either the 1 h (*F*_(4,25)_ = 49.85, *P* < 0.001) or 2 h (*F*_(4,25)_ = 17.12, *P* < 0.001) test day readings. However, there was a significant increase in streptozotocin-shortened FRL (dynamic vulvodynia) within 1 h (*F*_(4,25)_ = 27.38, *P* < 0.001) and 2 h (*F*_(4,25)_ = 10.08, *P* < 0.001) of PI-ME treatment at 200 mg/kg (*P* < 0.001, *P* < 0.05) and 300 mg/kg (*P* < 0.001, *P* < 0.01). The single test day positive control dose of gabapentin (75 mg/kg) alleviated both mechanical static and dynamic vulvodynia at the 1 and 2 h readings as evidenced by significant increases in FRT (*P* < 0.001, *P* < 0.01) and FRL (*P* < 0.001) versus the streptozotocin alone pretreated animals.

## Discussion

The antinociceptive activity of *P. incarnata* methanolic extract (PI-ME) was evaluated in mice using the hot plate test which is suitable for assessing centrally mediated acute phasic nociception [57] and the acetic acid induced abdominal constriction assay for tonic visceral nociception [58, 59]. Mice were selected as the species of choice in these specific tests because they are manifestly sensitive not only to opioid mediated effects but also to coexistent non-steroidal anti-inflammatory drug (NSAID) activity [51]. What is more, accumulating evidence indicates that GABAergic transmission plays a pivotal role in the inhibitory regulation of the nociceptive process, and the murine abdominal constriction assay as well as the hot plate test both detect dose dependent GABA agonist antinociception in this species [60, 61]. In both tests, diclofenac as a standard anti-inflammatory analgesic and PI-ME produced antinociceptive activity consistent with previous studies [16, 31, 62]. It was notable that the antinociceptive effect of PI-ME was reversed by the opioid- and GABA_A_- receptor antagonists, naloxone and pentylenetetrazole (PTZ) respectively, suggesting an involvement of opioidergic and GABAergic mechanisms in the mediation of the antinociception. Opioid agonists decrease pain transmission by activating descending nerve fibers from the periaqueductal gray and raphe nuclei supraspinally and also by inhibition of afferent nerve transmission by binding to pre- and postsynaptic opioid receptors within the spinal cord dorsal horn [63]. Furthermore, GABAergic neurons and receptors that are intercalated within the spinal cord and higher brain pathways are important for the origination, transmission, and modification of pain impulses in such a way that alteration of GABA transmission yields antinociception [64]. *P. incarnata* has been shown to modulate the activity of GABAergic and opioid systems [21] to produce central analgesic activity as evaluated by a reduced duration of paw licking in neurogenic and inflammatory nociceptive phases in the formalin test [31]. Due to a prevalence of GABA as a non-α-amino acid constituent of *P. incarnata* extract [65], several of its pharmacological effects have been ascribed to mediation via the GABA system. These include not only affinity for GABA_A_ but also GABA_B_ receptors in addition to modification of GABA uptake [66]. The antinociceptive effects of both GABA_A_ and GABA_B_ receptor agonists are known to involve activation or inhibition of other neurotransmitter or neuromodulator pathways [64] and it is evident that central GABAergic systems are involved in opioid-mediated analgesia [67]. Thus, it is possible that administration of GABA receptor agonists in combination with other agents may yield GABA receptor-related therapies for the treatment of acute and chronic pain [64].

The anxiolytic-like activity of PI-ME was assessed by the incidence of rears or steps climbed in the stair case test. The extract at a dose of 200 mg/kg significantly increased the number of climbed steps, although at a higher dose (600 mg/kg) it decreased this parameter. Similarly, the frequency of rears was diminished by the extract at all three doses tested and this outcome was blocked by PTZ. Anxiolytic-like activity has been shown to be associated with an increase in the number of steps climbed by mice whilst sedative activity is thought to be linked to a decrease in the frequency of rears [18] and this is the very reason why this paradigm was chosen in this species to evaluate *P. incarnarta*. Other studies have attributed an increased rearing incidence to an anxiety-like behavior and a decreased number of steps climbed to a sedative effect [68]. In conjunction with this, anxiolytic activity has been coupled with lower doses while sedative effects have been related to higher doses of plant extracts or reference drugs [69]. In this context, PI-ME displayed an anxiolytic-like effect at 200 mg/kg, while at 600 mg/kg it exhibited sedative activity. This was also confirmed in the open field test where it was observed that PI-ME decreased the number of lines crossed at doses of 400 and 600 mg/kg comparable to that of diazepam and these findings concur with the literature [17–19, 70]. Since PTZ reversed the anxiolytic-like and sedative actions of PI-ME, underlying GABA mediated mechanisms may well be implicated. In a selection of studies, the sedative and anxiolytic properties of *P. incarnata* have been attributed to benzodiazepine and GABA receptor mediated biochemical processes in the body [18, 19, 71, 72], binding to GABA_A/B_ sites and inhibition of GABA uptake being of particular consequence [66].

The modulatory effect of *P. incarnata* on GABAergic and opioid systems may provide some insight into its beneficial effect in various painful neuropathic conditions. Neuropathy induced hypersensitivity is known to involve disruption of tonic GABAergic transmission [73] and GABA agonists and metabolic inhibitors have been shown to be effective in various neuropathic nociceptive models [74–76]. Neuropathic pain has been reported to be refractory to opioids [77, 78]. However, several studies have shown that neuropathic pain can be attenuated by morphine and other μ-opioid receptor agonists [79–81] and these reports suggest that local μ-opioid receptors on the terminals of uninjured primary afferent nociceptive neurons are an essential target for alleviating mechanical allodynia. In the current study we have evaluated the methanolic extract of *P. incarnata* in a novel streptozotocin induced diabetic animal model of neuropathic pain established exclusively in rats [56]. The results showed that PI-ME (200 and 300 mg/kg) induced mechanical anti-allodynic activity exemplified by an increase in paw withdrawal threshold (PWT) and paw withdrawal latency (PWL) 1 and 2 h post treatment. Similarly, PI-ME also relieved dynamic vulvodynia by increasing the flinching response latency (FRL) although the extract was devoid of activity on the static component of vulvodynia. The intensity of the PI-ME dynamic anti-vulvodynia response was comparable to that of gabapentin which was used as a reference drug due to the fact that it has proven pain relieving effects in various neuropathic pain models [54]. Gabapentin also exhibits an established propensity to alleviate both static and dynamic components of allodynia and vulvodynia [56] and the current study corroborates this assertion. The present findings also indicate that the behavioural and antinociceptive effects of PI-ME involve GABAergic and opioidergic mechanisms because they were reversed by PTZ and naloxone respectively. Consequently, it might be inferred that analogous processes are implicated in PI-ME anti-allodynic/vulvodynic activity and this requires a direct focus of further study. In relation to this notion, Ingale and Kasture [31] suggested that opioidergic as well as the nicotinic cholinergic system are involved in the central analgesic activity of butanolic *P. incarnata* extract in the eye wipe test. This paradigm is used to study trigeminal pain because corneal nociceptive receptors have a large representation in the trigeminal ganglion through the ophthalmic branch of the trigeminal nerve [82]. Moreover, it has been suggested from radioligand binding studies that it is very unlikely that *P. incarnata* acts via the benzodiazepine-site of the GABA_A_-receptor [66]. In this connection, it has been postulated that GABA_A_ α1-sparing benzodiazepine-site ligands might constitute a class of analgesics suitable for the treatment of chronic pain syndromes [83]. Furthermore, there is considerable evidence implicating an important role for diminished GABAergic tone in the development of central sensitization and hyperalgesia in neuropathic pain models [84–86].

The phytochemical screening results of our study verify the presence of a preponderance of flavonoids as well as alkaloids in *P. incarnata* as described elsewhere [25, 87, 88]. Flavonoids are reported to be the major phytoconstituents of *P. incarnata* and include chrysin, vitexin, isovitexin, orientin, isoorientin, apigenin and kampferol [14, 30, 89]. These polyphenolic metabolites may play a role in the neuropharmacological activity of several plants [90–92] including *P. incarnata* [18, 93, 94]. Additionally, flavonoids have been reported to elicit an analgesic effect through opioid [95] as well as GABAergic systems [96] and have a beneficial role in relieving neuropathic pain conditions [97–99]. Some flavonoids like quercetin have also been identified in *P. incarnata* extract [100] and are believed to be effective in diabetes mellitus induced peripheral neuropathy [101, 102] the activity being mediated through an opioidergic mechanism [103]. The GCMS analysis in this study revealed that *P. incarnata* contains a predominance of the fatty acid amide 9-octadecenamide (also known as oleamide), which has hypnotic, analgesic, and anxiolytic actions [104]. Many of oleamide’s behavioural effects stem from its activity on various receptor systems including GABA_A_ [105–107], 5HT_1A_, 5HT_2A_, 5HT_2C_, 5HT_7_ [108–110], G-proteins [111], voltage gated sodium channels [107, 112] and CB_1_ receptors [113]. In this respect, oleamide enhances GABA receptor activity and specifically potentiates the peak chloride current when applied with GABA to benzodiazepine-sensitive GABA_A_ receptors [106]. The cannabimimetic action of oleamide resulting from its agonist action at CB_1_ receptors [110, 113] gives rise to cannabinoid antagonist reversible antinociception which is also sensitive to blockade by the GABA_A_ antagonist bicuculline [104]. It has been posited that endogenous fatty acid derivatives such as oleamide interact with endocannabinoids like anandamide in the modulation of pain sensitivity [114] which may well contribute to the inhibitory effect of *P. incarnata* on allodynia and vulvodynia observed in this study.

Other important constituents present in *P. incarnata* include hexadecanoic acid (palmitic acid), 3-hydroxy-dodecanoic acid, 2,3-dihydro-3,5-dihydroxy-6-methyl-4H-Pyran-4-one, and vitamin-E, that have strong antioxidant and neuroprotective activities and/or modulate the GABAergic system [115–119].

The modulation of GABAergic and/or opioidergic systems by *P. incarnata* reported in this study may constitute a proportion of the mechanisms implicated in the amelioration of diabetic neuropathy. Additional processes however, like a cannabimimetic action [110, 113, 114] cannot be ignored inasmuch as *P. incarnata* exhibits antihyperglycemic and hypolipidemic activities in streptozotocin induced diabetes mellitus [25] which would otherwise lead to neuropathic allodynia and vulvodynia [56]. Hyperglycemia and dyslipidaemia driven oxidative stress is a major contributor to reduced nerve function [120, 121] and diabetes mellitus is a major cause of peripheral neuropathy, commonly manifested as distal symmetrical polyneuropathy [122]. Furthermore, diabetes mellitus has been reported to be linked with vulvodynia either as an isolated symptom or as part of a constellation of other neuropathic abnormalities. Such neuropathic morbidity has been termed ‘diabetic vulvopathy’ and it profoundly affects patient’s quality of life and management needs in order to address the physical, psychological and relationship problems caused by the pain [123]. Our study showed that the methanolic extract of *P. incarnata* significantly alleviated only the dynamic component of vulvodynia which has been reported more likely to be provoked by contact with clothing among other triggers [124] and the cotton swab test is usually used to localize painful areas in vulvodynia [125].

## Conclusion

**Fig. 10:**
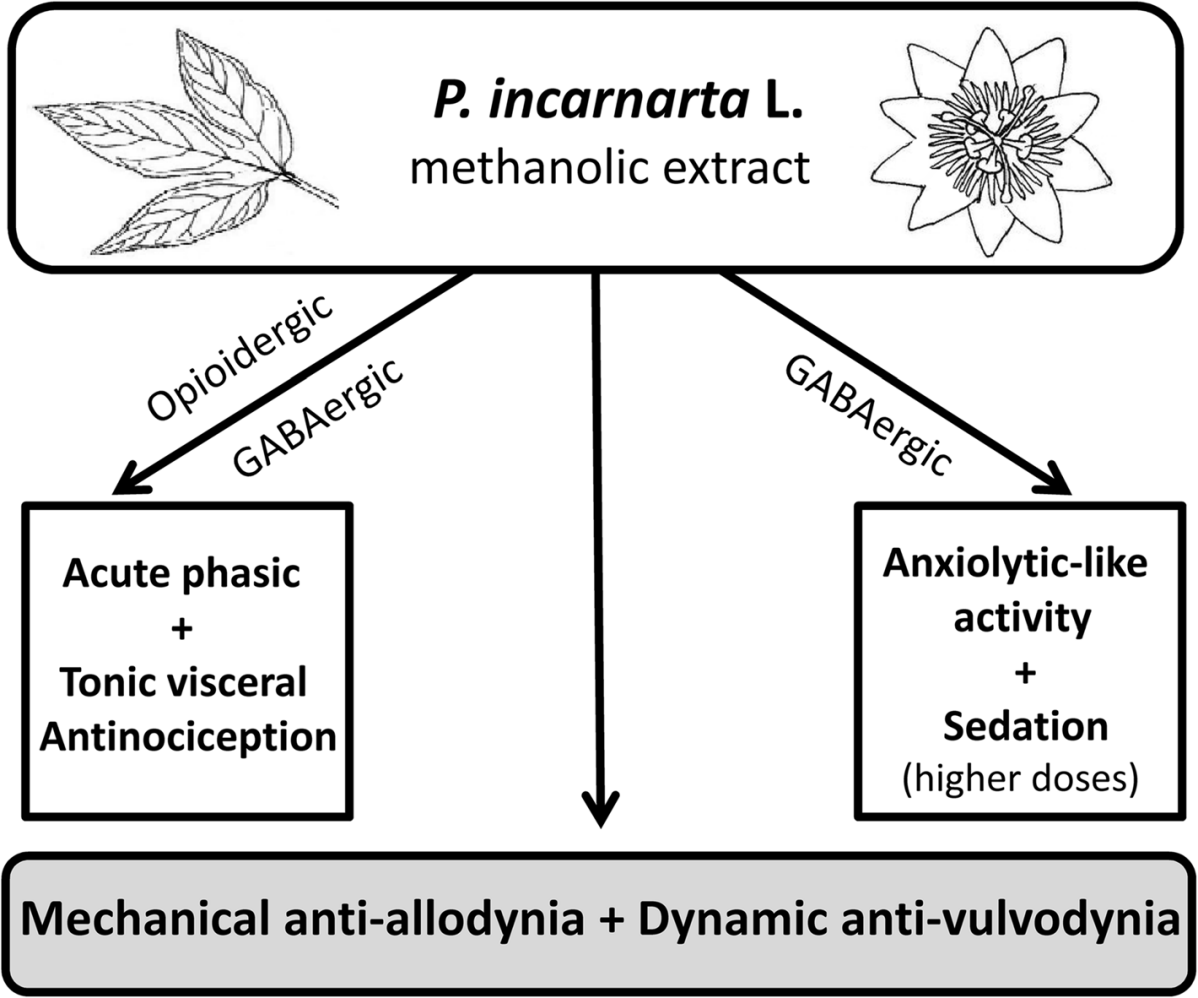
Scheme summarizing the anti-allodynic/anti-vulvodynic properties of *Passiflora incarnarta* plus it’s antinociceptive, anxiolytic-like and higher dose sedative activities

In conclusion, the methanolic extract of *P. incarnata* possesses peripheral and central phasic as well as tonic antinociceptive activity mediated through modulation of GABA_A_ and opioid receptors (GABAergic and opioidergic mechanisms shown in Fig. 10) which are disclosed by their naloxone and PTZ reversibility. The findings also manifest anxiolytic-like and higher dose sedative activity of the extract, resulting from GABAergic stimulation as indicated by their sensitivity to PTZ inhibition. The extract also exhibited significant mechanical anti-allodynic and dynamic anti-vulvodynic effects (Fig. 10) that may be attributable at least in part to the oleamide content and a cannabinoid-like action [110, 113, 114]. The outcomes from our study advocate an effectiveness of *P. incarnata* in the treatment of various neuropathic pain conditions. However, further studies are warranted in order to determine a more precise association between the active constituents responsible for the analgesic, anxiolytic and sedative effects of *P. incarnata* as well as the specific molecular mechanisms underlying its actions on allodynia and vulvodynia.

## Additional file

Additional file 1:
***Passiflora incarnata*** **plant, grown in the botanical garden of the Department of Pharmacy, University of Peshawar, Pakistan.** (DOCX 637 kb)
