# Supplementary material for: Passiflora incarnata attenuation of neuropathic allodynia and vulvodynia apropos GABA-ergic and opioidergic antinociceptive and behavioural mechanisms
Source: BMC Complement Altern Med. 2016 Feb 24;16:77. doi: 10.1186/s12906-016-1048-6 (PMC4765057; doi:10.1186/s12906-016-1048-6)
Supplement: Additional file 1: — Passiflora incarnata plant, grown in the botanical garden of the Department of Pharmacy, University of Peshawar, Pakistan. (DOCX 637 kb) [file 12906_2016_1048_MOESM1_ESM.docx]

**SUPPORTING INFORMATION**

***Passiflora incarnata* attenuation of neuropathic allodynia and vulvodynia apropos GABA-ergic and opioidergic antinociceptive and behavioral mechanisms**

Urooj Aman^1^, Fazal Subhan^1^*****, Muhammad Shahid^1^, Shehla Akbar^1^,

Nisar Ahmad^1^, Gowhar Ali^1^, Khwaja Fawad^1^, Robert D. E. Sewell^2^

**Affiliations:**

^1^Department of Pharmacy, University of Peshawar, Peshawar, Pakistan

^2^Cardiff School of Pharmacy and Pharmaceutical Sciences, Cardiff University, Cardiff CF10 3NU, UK

**Email addresses:**

Urooj Aman: ua_pharm@yahoo.com

Fazal Subhan: fazal_subhan@upesh.edu.pk

Muhammad Shahid: shahidsalim_2002@hotmail.com

Shehla Akbar: naina.akbar@yahoo.com

Nisar Ahmad: nisarahmadsatal@yahoo.com

Gowhar Ali: gohar.pharmacist@gmail.com

Khwaja Fawad: fawad.khwaja@yahoo.com

Robert D. E. Sewell: sewell@cardiff.ac.uk

***Correspondence:**

Dr. Fazal Subhan

Professor

Department of Pharmacy

University of Peshawar

Peshawar 25120

Khyber Pakhtunkhwa, Pakistan

Email: fazal_subhan@upesh.edu.pk

Phone: +92-91-9216750

Fax: +92-91-9218131


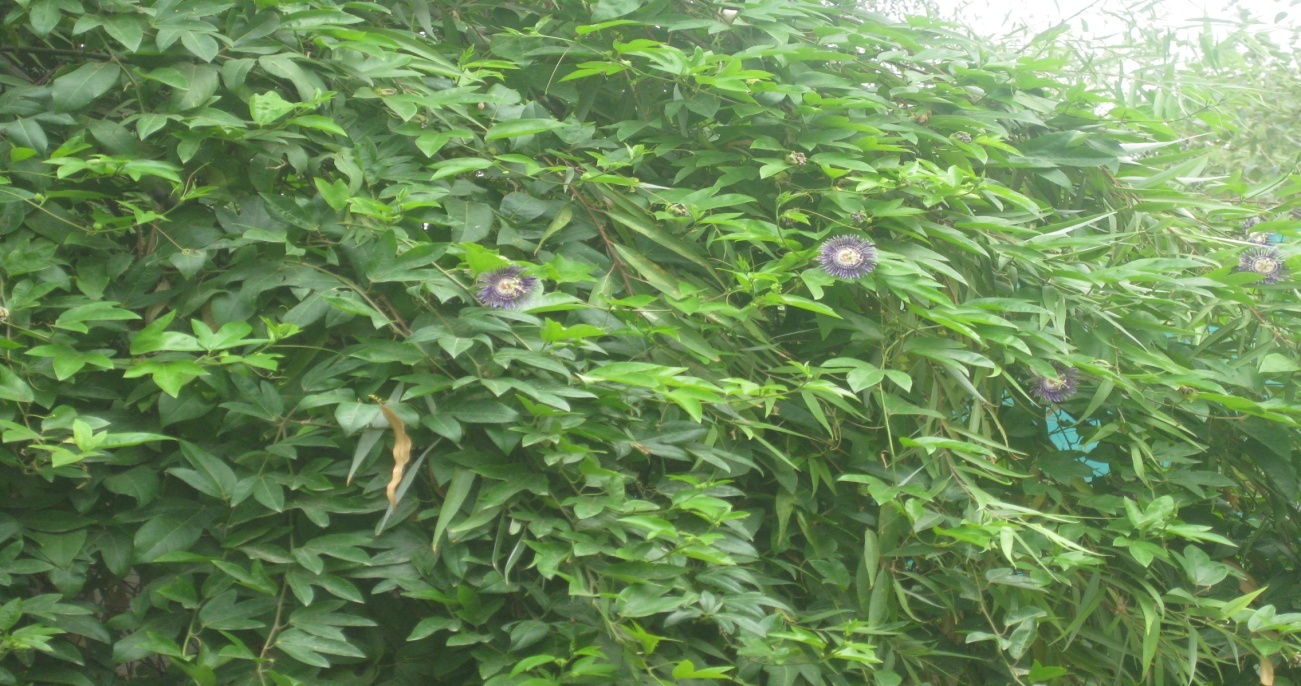


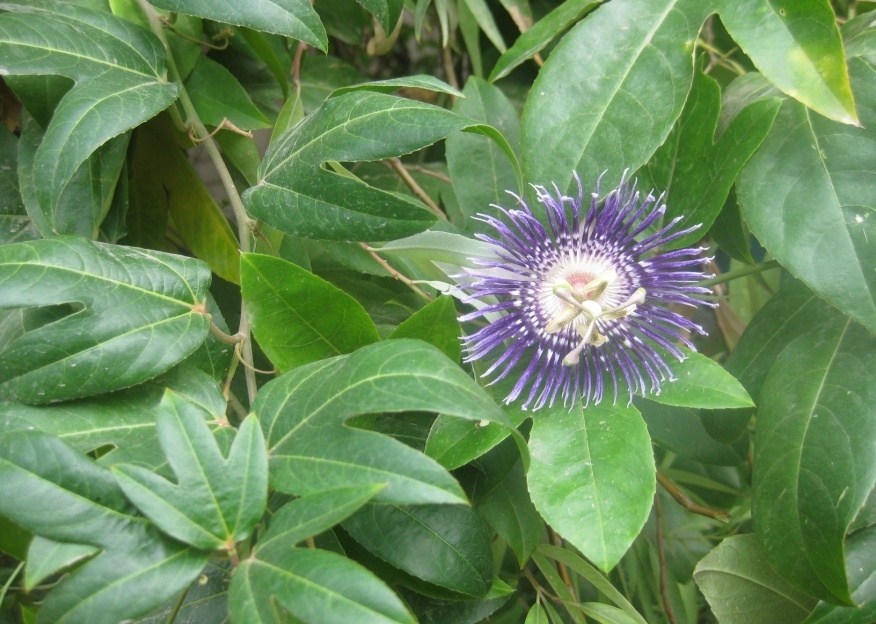


**Fig S1** *Passiflora incarnata* plant, grown in the botanical garden of the Department of Pharmacy, University of Peshawar, Pakistan.
